# Tebufenozide has limited direct effects on simulated aquatic communities

**DOI:** 10.1007/s10646-022-02582-y

**Published:** 2022-09-09

**Authors:** Christopher Edge, Leanne Baker, Emily Smenderovac, Shane Heartz, Erik Emilson

**Affiliations:** 1 Canadian Forest Service, Natural Resources Canada, Fredericton, NB Canada; 2 Biology Department, University of Waterloo, Waterloo, ON Canada; 3 Canadian Forest Service, Natural Resources Canada, Sault Ste. Marie, ON Canada

**Keywords:** Amphibian, Zooplankton, Microbe, Aquatic mesocosm, Tebufenozide

## Abstract

The use of insecticides to control undesirable pest species in forestry has undergone a shift from broad spectrum to narrow spectrum insecticides to reduce the risk of effects on non-target species. However, there is still risk of direct effects on non-target species as some insecticides function as hormone mimics, or through indirect pathways as the insecticide is broken down in the environment. Tebufenozide, an ecdysone hormone mimic, is the active ingredient in insecticides used in a variety of large scale pest control programs. An oft cited reason for the safety of Tebufenozide is that it is rapidly broken down in the environment by microbes. We investigated the potential non-target effects of two Tebufenozide formulations used in Canada, Mimic 240LV and Limit 240, on aquatic communities using an outdoor mesocosm experiment. We focus on direct effects on amphibian larvae (wood frog, *Rana sylvaticus*), zooplankton communities, and effects on biofilm and phytoplanktonic microbial communities that could arise from either direct toxicity, or from breaking down the insecticide as a nutrient and/or carbon source. There was limited evidence for direct effects on amphibian larvae or zooplankton communities. There were small but non-significant shifts in biofilm microbial communities responsible for nutrient cycling. Beta diversity in the plankton community was slightly higher among tanks treated with insecticide indicating a community dispersion/disbiosis effect. Overall, we found limited evidence of negative effects, however, subtle changes to microbial communities did occur and could indicate changes to ecosystem function.

## Introduction

Insecticides are commonly used around the world in various integrated pest management programs. A tenant of integrated pest management is to use selective, narrow spectrum insecticides due to their high efficacy and low risk of non-target environmental effects (Casida and Quistad 2004; Castle and Naranjo 2009; Holmes and MacQuarrie 2016). Some selective, narrow spectrum insecticides disrupt the endocrine system by inhibiting the action of hormones which are specific to certain taxa, or alter the normal regulatory function of the immune, nervous, and endocrine systems (Crisp et al. 1998; Dhadialla et al. 1998; Casida and Quistad 2004). There is potential for hormone mimicking insecticides to have non-target effects because the endocrine system is highly conserved across multiple taxa (McLachlan 2001). Further, many insecticides are broken down by microbes in the environment, and could lead to changes in microbial community composition and function, which could indirectly alter ecosystem services.

Tebufenozide has been registered to control forest pests in Canada since 1996 and is used for the control of spruce budworm (*Choristoneura fumiferana*) and hemlock looper (*Lambdina fiscellaria*) (West et al. 1997; Holmes and MacQuarrie 2016). Tebufenozide is thought to have limited direct non-target effects because the active ingredient mimics the insect molting hormone 20-hydroxyecdysone causing premature and incomplete molting of lepodopteran larvae (Smagghe and Degheele 1994). Currently, aerial application of Tebufenozide accounts for approximately 10% of insecticide application in Canadian forestry and is used as part of the Early Intervention Strategy for the control of spruce budworm in Eastern Canada (MacLean et al. 2019). The major breakdown and detoxification pathway of Tebufenozide in the environment is mediated through microbial activity (Sundaram 1994; Sundaram et al. 1996).

A significant amount of prior work investigating non-target effects found no treatment effects at environmentally realistic exposures on zooplankton communities (Kreutzweiser et al. 1998; Kreutzweiser and Faber 1999), phytoplankton abundance (Kreutzweiser and Thomas 1995), macroinvertebrates (Kreutzweiser et al. 1994), aquatic arthropods (Song et al. 1997) amphibian embryo hatching (Pauli et al. 1999), and soil invertebrates (Addison 1996). The hormone, 20-hydroxyecdysone, which Tebufenozide mimics, or hormones of similar structure are produced by a variety of insect taxa (Smagghe and Degheele 1994; Hahn et al. 2001). In some of these taxa, exposure to Tebufenozide can cause improper molting (Hahn et al. 2001). Potential non-target effects could occur in other taxa, such as amphibians, that employ similar hormones during development.

The growth and development of amphibian larvae is controlled by the hypothalamus-pituitary-adrenal (HPA) axis. Amphibian metamorphosis is largely under the control of the thyroid hormones, thyroxine (T4) and triiodothyronine (T3), and the stress hormones, corticosterone and alderstrone (Denver et al. 1998; Boorse and Denver 2003; Buchholz and Hayes 2005; Denver et al. 2002; Denver 2009). Within amphibians, and other vertebrates, the HPA axis and its effects on development are highly conserved (Rose 2005; Martin et al. 2011). Thyroid hormones are similar in function to ecdysone moulting hormones, including 20-hydroxyecdysone, but they differ in structure (Ollikainen et al. 2006). Despite the fact that the structure of ecdysone and thyroid hormones differ it is possible that hormone mimicking insecticides could have effects on survival, growth, and development of amphibian larvae. As amphibians are declining around the world (Stuart et al. 2004) and exposure to pesticides is one of several causal mechanisms (Hayes et al. 2006; Tang et al. 2021), understanding risk from common and wildly used insecticides, such as Tebufenozide, is extremely important.

Despite the fact microbial communities contribute much of the primary production in lentic systems, and are involved in nutrient cycling, decomposition, and oxygen generation the effects of Tebufenozide on their composition and function has not been explored in detail (Sundaram 1997a; Gómez De Barreda Ferraz et al. 2004). Direct toxic effects of Tebufenozide on aquatic microbial communities are possible, but unlikely due to the specificity of the active ingredient. However, macroecological changes that occur as the microbial community breaks down the active ingredient (Sundaram 1997b, c) are possible. Artificial enhancement of certain species over others can enable the occupation of niches that might otherwise be performing important ecosystem functions (e.g., allow non-phototrophic organisms to dominate). Microbial communities are also influenced by changes to the ecology of a system, and so changes to microbial communities could occur as an indirect response to changes in populations of other organisms following application (Sundaram 1997a; Estrela et al. 2021).

In the aquatic environment, both phytoplankton and substrate biofilm microbes could be affected by exposure to pesticides. Previous work found no effect of exposure to Tebufenozide on the abundance of phytoplankton (Kreutzweiser and Thomas 1995; Kreutzweiser et al. 1998). However, recent techniques allow for testing effects on changes to communities and not just abundance. Past work has explored potential effects on soil microbial communities in an agricultural context (Walters et al. 2015), but no work has focussed on aquatic planktonic and biofilm communities in a forestry context.

The goal of the present study is to evaluate the effects of a widely used insecticide in Canadian forestry, Tebufenozide, on aquatic communities. Specifically, we expand the knowledge on possible non-target effects by testing for response of amphibian larval development, zooplankton communities, and microbial (plankton and biofilm) communities.

## Methods

The experiment was conducted at the Forest Aquatic Mesocosm Array located at the Acadia Research Forest in New Brunswick. The aquatic mesocosms are designed to mimic small forested wetlands where exposure to Tebufenozide could occur from large scale insect control programs, such as those for the control of eastern spruce budworm. We tested two Tebufenozide insecticide formulations that are in use in Canada, Limit 240 (Spray Industries, Saint John, New Brunswick; Pest Control Products Act Registration Number 32535) and Mimic 240 LV (Valent Canada Inc, Guelph, Ontario; Pest Control Products Act Registration Number 24502), at two exposure concentrations and an untreated control. Each of the insecticides were tested at an Expected Environmental Concentration (EEC), the concentration in a 15 cm deep wetland with no intercepting vegetation oversprayed at the maximum label application rate (0.046 mg/L, rounded to 0.05 mg/L), ½ of the EEC (0.025 mg/L), and a control. The EEC concentration should be considered a worst case exposure scenario. Each treatment was replicated six times. Both of the insecticide formulations are used in the Early Intervention Strategy for the control of Spruce Budworm in Eastern Canada (MacLean et al. 2019).

The experiment was conducted using 1100L Rubbermaid cattle tanks (diameter: 161 cm, height: 63.5 cm) buried approximately 2/3 in the ground. Each tank has a screened overflow valve to maintain water levels at 1000 L and a removable shade cloth. On 17 May 2019 tanks were filled with 1000 L of well water and 25 L of leaf litter collected from under American beech trees (*Fagus grandifolia*) in the surrounding forest was added to create realistic substrate. Tanks were left for 2 days to allow the leaf litter to sink and for water temperature to rise. On 19 May 2019, zooplankton were collected from a nearby wetland by taking ~500 hauls with a students plankton net. Hauls were pooled, mixed and diluted to a total volume of 35 L. After mixing and dilution, 1 L of concentrated zooplankton were added to each tank. In addition, three replicate standardized leaf packs (8–10 g of pre-weighed speckled alder leaves (*Alnus incana*) in a 0.5 mm mesh pack) were added to each of the tanks. The leaf packs served as standardized substrate for the colonization by microbial biofilms, with leaf material used for eDNA extraction (for determination of community composition) and extracellular enzyme assays (for determination of effects on nutrient cycling functions).

On the same day that the zooplankton and microbial leaf packs were added (19 May 2019) 20 wood frog (*Lithobates sylvaticus*) egg masses were collected from wetlands in the surrounding forest and placed in small containers until hatching. When the tadpoles reached Gosner stage 25 (Gosner 1960), on 29 May 2019, the larvae from all egg masses were mixed and 125 larvae were added to each tank. During the experiment tadpoles grazed algae that grew on the sides of the tanks and on the leaf litter, no supplemental feeding occurred. No water changes were performed.

Stock dilutions of both insecticides were made by diluting the formulated insecticide into well water so that 20.83 ml (EEC) or 10.42 ml (1/2 EEC) of diluted insecticide could be added to each tank. To ensure all tanks experienced the same disturbance, 20 ml of well water was added to each of the control tanks. Additions were made by pouring the diluted insecticide or water evenly over the surface of the tanks 12 days after the tadpoles were added on 10 June 2019. Depth integrated water samples were collected from each tank one day after application to confirm treatment concentrations. Samples were collected in amber bottles, placed in a cooler on ice, and immediately frozen at the end of the day. Tebufenozide concentration was determined by LC-MS/MS at the University of Guelph Agriculture and Food laboratory.

### Amphibian analyses

The experiment was terminated when the tadpoles began to reach Gosner stage 42, emergence of the forelimbs, 35 days after treatment (DAT). On DAT 35 tanks were searched for surviving larvae by carefully draining and removing all the leaf littler. All tadpoles were removed from the tanks and euthanized by emersion in MS-222 in the field. In the laboratory the development stage of tadpoles was determined according to Gosner (1960) and the snout-vent length of animals was measured. Percent survival was calculated from the number of animals remaining in each mesocosm at the end of the experiment and analyzed with ANOVA, data were arcsin squareroot transformed prior to analyses. Development stage was analyzed using a Linear Mixed Effect Model (LMM) with treatment as a factor and tank as a random block. Snout-vent length was first analyzed by testing for a relationship between development stage and snout-vent length with a LMM with development stage as a fixed effect and tank as a random effect. Snout-vent length was then analyzed with a LMM with treatment as a categorical fixed effect, development stage as a continuous fixed effect, and tank as a random effect. Development stage was included as a fixed effect because it was significantly correlated with body size.

### Zooplankton

The zooplankton community was sampled 30 days after treatment application. Zooplankton were sampled by pooling three hauls using a 15 cm Student plankton net from each tank. Zooplankton were identified to species, length measured and weight estimated based on established length-weight regressions (Girard et al. 2007). Total biomass was analyzed using a factorial ANOVA with treatment as a factor. The zooplankton community was analyzed with non metric multidimensional scaling (NMDS) and a PERMANOVA using Bray Curtis dissimilarity distance and 999 permutations.

### Biofilm microbial analyses

Leaf packs were collected on DAT 7, 14, and 21 to capture the short-term responses to Tebufenozide additions. On each sample day one leaf pack was removed, placed in a labelled container and immediately kept on ice until transfer to a freezer (−20 °C) within 8 h.

Extracellular enzyme activities were measured for enzymes involved in cycling of carbon (β-glucosidase and xylosidase), nitrogen (N-acetylglucosaminidase), and phosphorous, (phosphatase). All enzyme activities were performed under controlled conditions using MUB-tagged substrates. Samples were stored at −20 °C prior to analysis via existing protocols (Saiya-Cork et al. 2002; Findlay 2007). DNA extractions were performed on homogenized leaf material, which were then sequenced using primers targeted for Fungal ITS (ITS9F, ITS4R) and Bacterial 16S (515F, 926R) regions on the Illumina MiSeq platform. Data was processed into ASVs (Amplicon Sequence Variants) using the MetaWorks pipeline v1.4.1 (Porter and Hajibabaei 2020). Samples with <1000 reads were removed. Taxonomic assignments were applied as per the appropriate cutoff values outlined in the MetaWorks pipeline. Taxonomy of 16S data was assigned using the rdp 2.1.3 database included with MetaWorksv1.4.1. ITS sequences were classified with the UNITE v8.2 ITS reference set database (Kõljalg et al. 2019). Functional pathways were assigned through picrust2 (Douglas et al. 2020).

A non-parametric community richness estimator, the Chao1 index, was used to estimate community diversity, as this index has been shown to work well with datasets skewed by many low-abundance counts (Chao 1984). Compositional analyses were performed on data transformed using centered log ratio and consisted of partial RDA with Tebufenozide treatments and amounts as constraining variables, and DAT as a control variable. ALDEx2 was used to perform compositional analyses of individual taxa. ALDEx2 glms were performed for each targeted amplicon (i.e., 16S, ITS) with Tebufenozide treatment and an interaction term including treatment amount and DAT. Changes in picrust pathways and enzyme activities were assessed using the lm function with Tebufenozide and an interaction treatment including DAT and amount, these results were assessed at an α of 0.05 and a Bonferroni corrected α of 0.05.

### Plankton microbial analyses

Plankton were sampled by taking 5, 1 L depth integrated samples from random locations in each tank. The five samples were pooled and a 1 L composite sample was taken, immediately placed on ice, and frozen within 8 h of collection. In the laboratory samples were thawed and a known volume of water was filtered onto sterilized 500 µm filter paper using suction filtration. Equipment was rinsed with tap water and sterilized with ethanol between samples.

To compare the diversity of prokaryotes (bacteria, including cyanobacteria) and eukaryotes (diatoms and green algae), genomic DNA extractions were performed on filtered plankton samples using DNeasy extraction kits, following manufacturers recommendations (QIAGEN, Toronto, Ont). 16S and 18S small subunit rRNA gene amplicon sequencing was performed using universal primers (16S; (Walters et al. 2015)) or phototrophic-specific primers (18S; (Bradley et al. 2016)) for bacterial and algal communities respectively. Amplicons were sequenced using the Illumina MiSeq platform (V2 2 × 250 kits). After sequencing, the raw FASTQ data were processed into amplified sequence variants (ASVs) using DADA2 (Callahan et al. 2016) and QIIME2 (Bolyen et al. 2019). Taxonomy was assigned using a naive Bayesian classifier trained against SILVA v132 (Quast et al. 2013). Using the phyloseq package in R (McMurdie and Holmes 2013), reads were normalized using median sequencing depth, filtered to remove very rare taxa (<0.1%) and non metric multidimensional scaling (NMDS) was performed on calculated UniFrac distances (a distance metric that incorporates the relative relatedness of community members based on phylogenetic distance). The community richness estimator, Chao1, was used to estimate community diversity.

### Statistical packages

All statistics were performed in R using the base (Core Team 2021), lme4 (Bates et al. 2015), vegan (Oksanen et al. 2020) packages. Metabarcoding data were analyzed in R using data transformation functions found in the tidyverse package (Wickham et al. 2019). Figures were produced using the ggplot2 package (Wickham 2016). All means are reported ±SD.

## Results

### Exposure

Tebufenozide was detected in one control tank at a concentration of 0.0004 mg/L (more than 60× below that of the lowest treatment levels), and contamination likely occurred when the water samples were collected from each tank. The mean concentration in the Mimic EEC and Limit EEC treatments was 0.065 ± 0.0059 and 0.052 ± 0.00477 mg/L, respectively. Mean concentrations in the Mimic ½ EEC and Limit ½ EEC treatments were 0.0292 ± 0.00488 and 0.0267 ± 0.00121 mg/L respectively. Measured values approximate nominal target concentrations of 0.05 and 0.025 mg/L for the EEC and ½ EEC treatments.

In one of the control tanks 140 wood frog larvae were captured at the end of the experiment which is more than the number of animals nominally added (125) at the start of the experiment. This tank was removed from all analyses.

### Amphibians

**Fig. 1:**
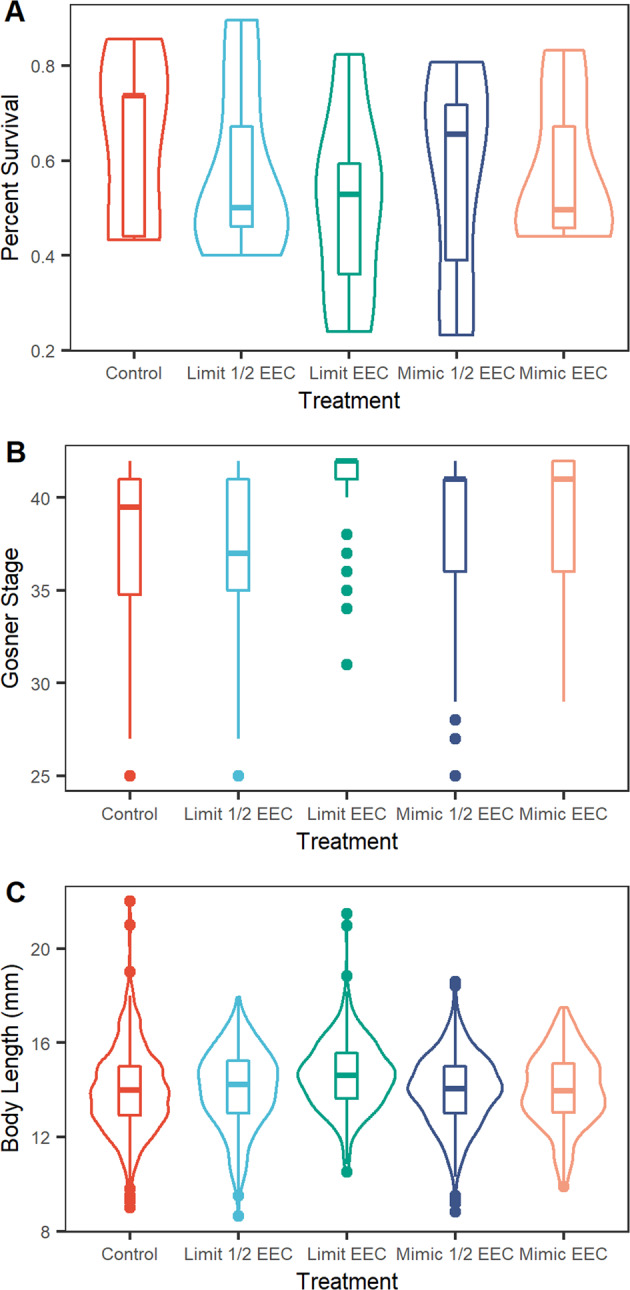
**A** Tadpole survival, (**B**) Gosner stage, and (**C**) Body Length in the control and different Tebufenozide treatments. Tadpoles in the Limit EEC treatment were more developed (**B**) than those in all other treatments

Overall, the mean survival in all tanks was 58.9% (±19.1%) and it did not differ among the treatments (*F* = 0.31, df = 4,24, *p* = 0.87) (Fig. 1A). Development stage at the end of the experiment did not differ among any of the treatments (*p* > 0.05) (Fig. 1B). Tadpole body length was positively related to developmental stage (*p* < 0.001) and there was no difference in tadpole body length between any of the treatments (*p* > 0.05) (Fig. 1C).

### Zooplankton

**Fig. 2:**
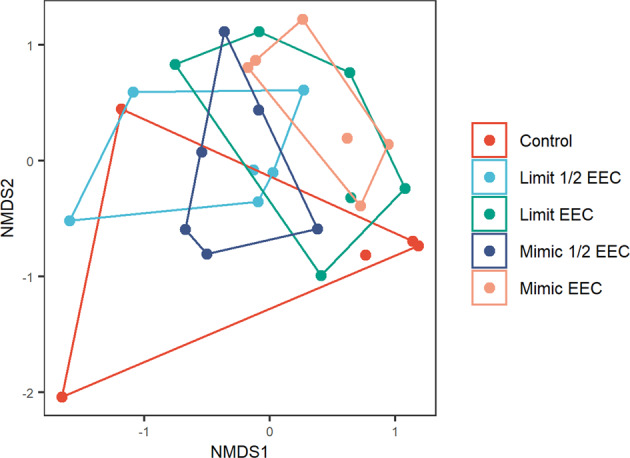
NMDS plot showing Zooplankton community structure in five Tebufenozide treatments

Across all tanks a total of 6 species of zooplankton were found (Table 1). The average number of species in each tank was 4 (±1.16), and there was no difference in species richness (*F* = 0.316, df = 4,24; *p* = 0.86) or total biomass (*F* = 1.67, df = 4,24, *p* = 0.190) among the treatments. A solution to the NMDS was reached after 20 runs and with a stress value of 0.15. Of the six detected species all but one contributed significantly to the axis (Table 1). There were no differences in the zooplankton communities among the different treatments (*F* = 1.59, df = 4,24, *p* = 0.071, *R*^2^ = 0.209) (Fig. 2). Together, the four Tebufenozide treatments occupy a slightly different space in the NMDS plot, but this difference appears to be driven by one control tank which had the lowest species richness (1) of all the replicates.

**Table 1:** List of Zooplankton species detected and significance value for contribution to NMDS plot

Species	Number of tanks	*P* value for contribution to NMDS
*Acanthocyclops vernalis* complex	16	0.002
*Ceriodaphnia sp*.	15	0.013
*Cyclopoid copepodid*	26	0.001
*Cyclopoid Nauplius*	24	0.044
*Scapholeberis mucronata*	26	0.001
*Simocephalus serrulatus*	11	0.572

### Biofilm microbes

**Fig. 3:**
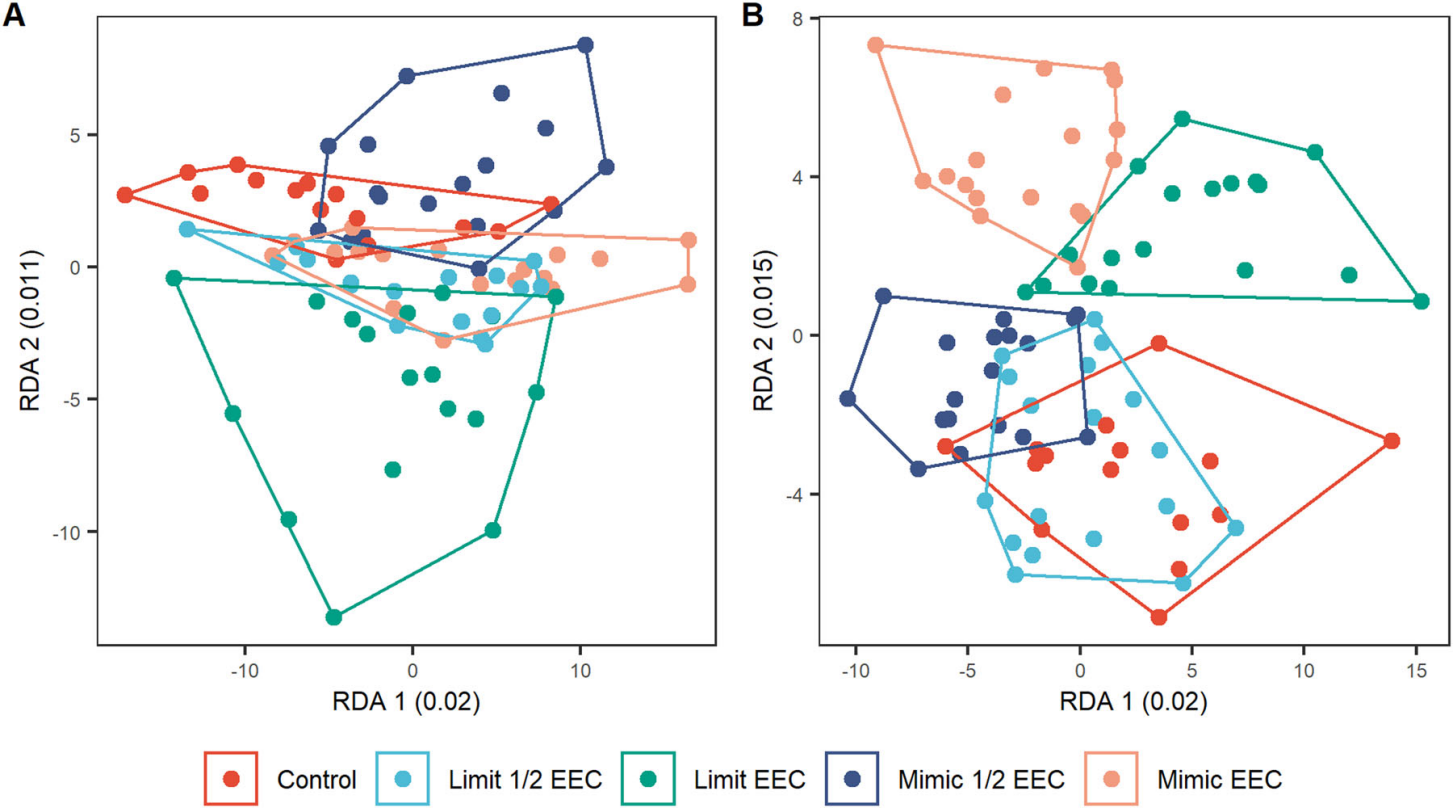
RDA of centre-log ratio transformed compositional community structure for biofilm microbial (**A**) 16S and (**B**) ITS2 metabarcoding assemblages. The total explained variance shows the entirety of the constrained variance for these RDAs, including the two axes plotted

There were no significant differences in substrate fungal or bacterial diversity due to Tebufenozide treatment, though there were significant increases in bacterial diversity over time as the leaf packs slowly accumulated biofilm communities. Although the RDA suggests some organisms responded to the EEC treatments (Fig. 3), no single ASV had an RDA axis score >0.42 with most having generally weak (<0.2) scores on constrained axes. While there is some visual separation on RDA axes, the overall low variance explained by the partial RDA, and the lack of high species scores on those axes show that any differences were small and corresponded to small changes amongst many groups.

Identified Genus of ASV with RDA axes scores greater than 0.2 included *Altererythrobacter, Asticcacaulis, Bdellovibrio, Chlorophyta, Clostridium sensu stricto, Devosia, Dioszegia, Ferruginibacter, Flavobacterium, Flectobacillus, Formivibrio, Gemmobacter, Geomonas, Hannaella, Holophaga, Kondoa, Legionella, Novosphingobium, Paludibacter, Pedobacter, Pelosinus, Pilidium, Pseudomonas, Shinella, Sphingobium, Sphingomonas, Spirochaeta, Spirosoma, Uliginosibacterium* and*, Vishniacozyma*. All of which have the capability of using complex carbon sources, or autotrophic metabolism, which may indicate increased competitiveness of species that breakdown Tebufenozide.

Aldex glm corroborated the results of the compositional RDA analysis, in that there were no ASV significantly associated with Tebufenozide treatment after Benjamini-Hochberg corrections were applied (Table 2). Organisms that were associated with the treatments before corrections were investigated graphically and were all of either low abundance, or were inconsistent within treatment groups. Picrust was used to create predicted metabolic profiles of the samples. Overall, 430 pathways or four enzyme activities studied, of which 20 were significant at critical values of 0.05, however none were significantly associated with treatment after Bonferonni correction or had strong, consistent patterns upon visual examination. Additionally, there were no significant effects of Tebufenozide treatment on enzyme activities for N-Acetyl glucosaminidase, Phosphatase, or Xylosidase (*p* > 0.05).

**Table 2:** Number of ASV with compositional variance changes responding to treatment effects from the biofilm microbial analyses

Set	Bacterial (16S)	Fungal (ITS)
Number of ASV with significant Treatment effect at *α* = 0.05	54	6
Number of ASV with significant Treatment effect after Benjamini-Hochberg correction	0	0
Total number of ASV	6637	5005

### Plankton microbes

**Fig. 4:**
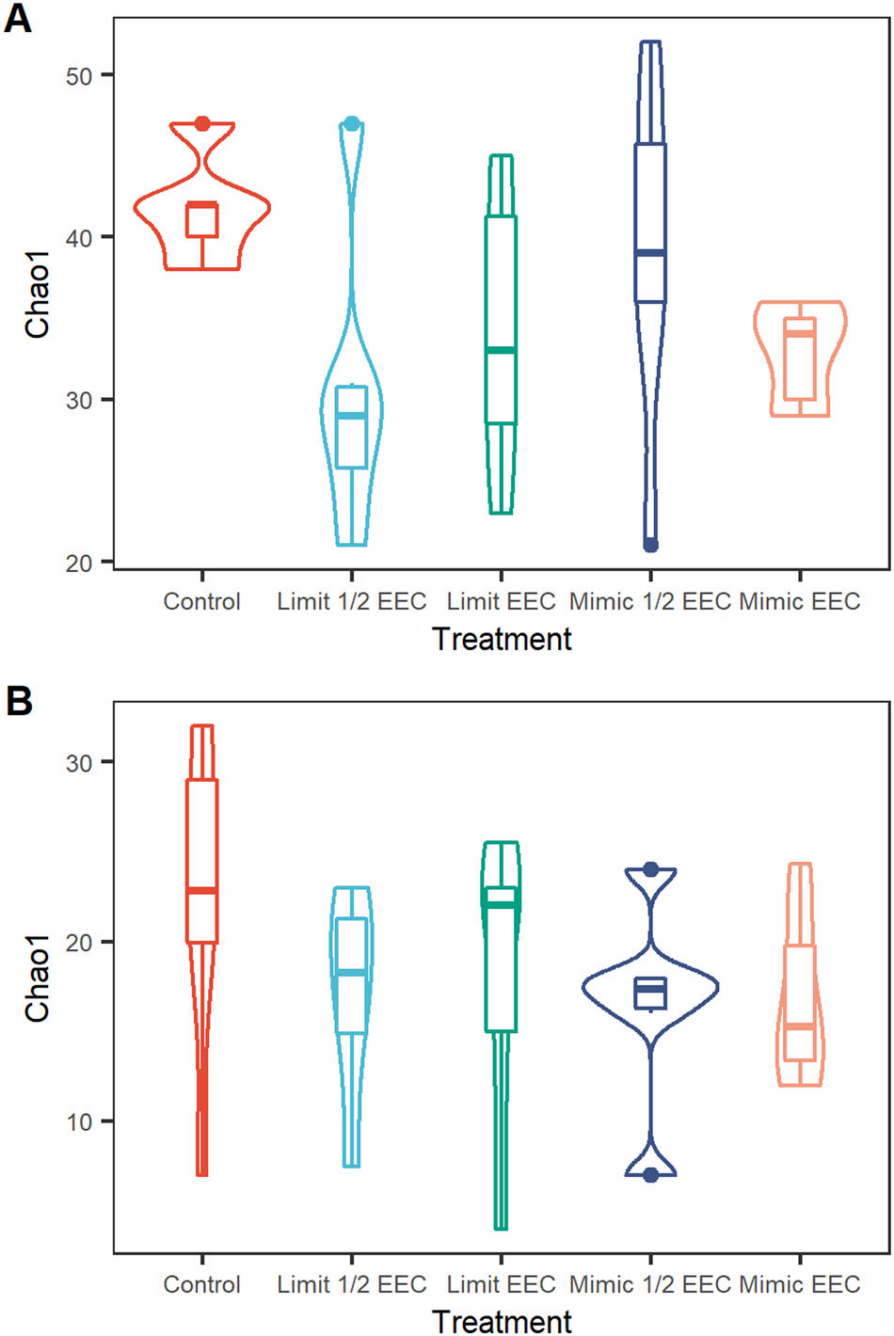
Alpha diversity of plankton (**A**) 16S Bacterical communities and (**B**) 18S Algal communities in mescosms treated with Tebufenozide pesticides

**Fig. 5:**
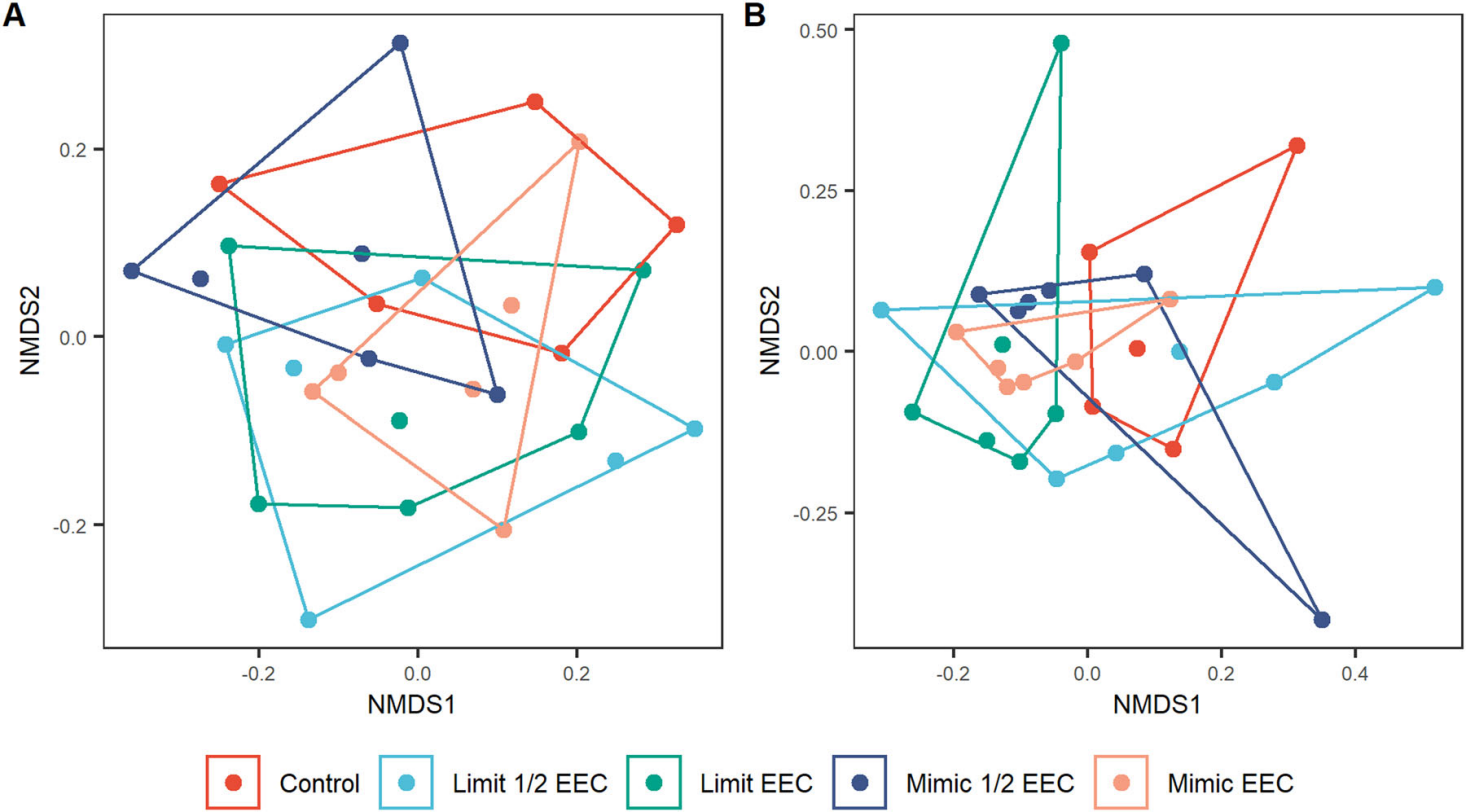
Ordinations of the effects of treatment types on plankton (**A**) 16S Bacterial community composition and (**B**) 18S Algal community composition. Convex hulls contain each combination of chemical type × concentration applied

Illumina sequencing identified 885 unique bacterial (16S), and 226 unique algal taxa (18S) in the plankton communities. Both the bacterial and algal plankton communities were less diverse on average in all treatment categories than in the reference mesocosms (Fig. 4), although the changes in diversity did not follow a dose-dependent response. For the bacterioplankton community a solution to the NMDS was reached after 20 runs with a stress of 0.17. Overall, the bacterial components of the plankton community did not differ among treatments on either axis (axis 1: df = 2,24, *p* = 0.53; axis 2: df = 2,24, *p* = 0.28). The bacterioplankton community in reference mesocosms had very similar taxonomic composition (Fig. 5A). The bacterial community appeared to respond more the treatment type than the concentration level. The application of the Limit formulation at both treatment concentration resulted in an apparent dispersion effect (or dysbiosis) in the bacterial community, where the composition shows greater variability among replicates than that observed in the control mesocosms. The effect of the Mimic formulation on the bacterial community was more discrete, where both the EEC and half EEC appeared to shift the bacterial community in the same general direction, although the convex hulls of these two treatments still showed some overlap with the reference mesocosm bacterial community composition.

In the algal components of the plankton community a solution to the NMDS was reached after 20 runs with a stress of 0.17. Overall, the algal community did not differ on either of the axis (axis 1: df = 2,24, *p* = 0.57; axis 2: df = 2,24, *p* = 0.94). In general the effect of concentration appeared to have a more consistent effect than the brand of Tebufenozide-containing pesticide (Fig. 5B). Mesocosms treated with EEC concentrations of pesticides has nearly no overlap of convex hulls (representing their community composition) with the control mesocosms. Half EEC doses of Tebufenozide pesticides resulted on algal communities similar to the control mesocosms (overlap of convex hulls), although there was a greater diversity among the algal communities of the half EEC treated mesocosms (larger convex hulls than control).

## Discussion

Narrow spectrum insecticides, such as Tebufenozide, are used extensively when they are effective and pose low risk to non-target taxa. However, their widespread use increases the likelihood non-target species and communities could be exposed raising the need to study potential effects across a wide range of ecosystems. We found limited evidence of effects on survival, growth or development of a common North American amphibian tadpole, the wood frog. Tadpoles in the Limit EEC treatment were more developed, but not statistically significant, than in the control treatment at the end of the study. If Tebufenozide mimicked hormones responsible for development in tadpole larvae then accelerated development would be expected (Sanchez-Bayo 2011). However, a similar response would be expected in both insecticide formulations because they contain the same active ingredient and the exposure concentration for both formulations was the same, therefore any difference is unlikely to be treatment related.

Many zooplankton species are quite sensitive to environmental changes and zooplankton communities are used as indicators of environmental change (Attayde and Bozelli 1998; Jeppesen et al. 2011). In the present study we found no differences in the zooplankton communities among the treatments. The communities that established in the mesocosms consisted of 6 species and were relatively simple. Other studies on natural wetlands in the region this study was conducted found individual wetlands have between 3 and 12 species from a total species pool of 18 (Baker et al. 2016). The communities we established in the mesocosms are therefore representative of those in the area, but were missing some potential species. Our results are comparable to previous work that found no effect exposure to Tebufenozide on zooplankton communities (Kreutzweiser et al. 1998; Kreutzweiser and Faber 1999).

Algal species have demonstrated some level of toxicity to Tebufenozide (Gómez De Barreda Ferraz et al. 2004) at concentrations (0.017–5.9 mg/L) near those tested in the present experiment (0.025 and 0.05 mg/L). Within the bacterial and algal components of the plankton community we observed a slight dispersion effect, increased size of convex hull, in some of the pesticide treatments. Community dispersion is often attributed to stochastic, rather than deterministic effects, and thought to be common in microbial communities (Zaneveld et al. 2017). In the bacterioplankton community we saw evidence that the formulation of the pesticide was more important than the application concentration, whereas in the algal community, the higher concentration of each pesticide had a greater effect than that of the pesticide identity. Both pesticides contain Tebufenozide, whereas the other ingredients included in the formulation differ, and as such, it may be that the planktonic algal community is affected by the presence of the Tebufenozide itself, and the bacterial community may be affected more by the proprietary ingredients, which differ between the formulations. We can only speculate on the nature of these ingredients, due to the proprietary nature of pesticide formulations. While we cannot claim a particular ingredient is responsible, simple carbohydrates such as glycerol and glycerin are common ingredients and easily utilized by chemoheterotrophic microorganisms. In oligotrophic conditions, similar to those established in the mesocosms at the start of the experiment, addition of simple carbohydrate may increase the competitiveness of chemoheterotrohic microorganisms and shift community structures (Estrela et al. 2021). Overall, the observed changes were small and in need of further study to confirm.

A small amount of variance (5%) in the biofilm microbial community could be attributed to the Tebufenozide treatments. That most of the organisms that loaded strongly on the RDA axis (>0.2) were either heterotrophs with some capability of breaking down complex carbon, or autotrophs is somewhat indicative of small changes occurring as the microbial community uses Tebufenozide as a nutrient or carbon source. However, these were small, statistically insignificant changes and no treatments had significant responses in any ASVs from either ITS or 16s metabarcoding indicating that exposure to Tebufenozide did not lead to an overall change in the microbial community. The differences between the groups are very small as shown by the limited variation explained by the treatment factor. It may be that much of the Tebufenozide is degraded in the water column (Sundaram 1997b), and that a very small amount is left to influence biofilm communities, which have other, easily accessible carbon sources to use. These community changes do not appear to translate top changes in ecosystem function, as evidenced by the lack of quantifiable functional differences from enzyme analyses.

The DT50 for Tebufenozide in aquatic environments has been estimated at 32–35 days (Kreutzweiser et al. 1998) and 52–115 days in litter and soil (Sundaram 1997b). Our experiment lasted for 35 days, and measured concentrations 24 h after application was made approximated the target exposures (0.05 and 0.025 mg/L). It is therefore likely that organisms in the tanks were exposed to concentrations that approximated nominal target concentrations for the duration of the experiment. Monitoring of aqueous exposure concentration for the duration of the experiment, rather than immediately after application, would help elucidate any time dependant effects. Measured aquatic concentrations, as part of operational monitoring, found maximum concentrations of 0.0003 mg/L in river systems (Owens Pers Comm). Therefore, the conditions tested herein can be considered an unlikely, worst case exposure scenario from operational use of the two Tebufenozide insecticides commonly use for the control of spruce budworm in Eastern Canada.

## Conclusions

Overall we found limited evidence of effects on amphibians, zooplankton, or microbes from exposure to two different insecticides used in Canada that contain Tebufenozide as an active ingredient. Within the microbial communities there were small insignificant community shifts that could indicate slight changes in community composition. Tebufenozide is broken down in the environment by microbes, but the added nutrient source does not appear to lead to changes in overall community composition or function.
